# Supplementary material for: Recovering What Matters: High Protein Recovery after Endotoxin Removal from LPS-Contaminated Formulations Using Novel Anti-Lipid A Antibody Microparticle Conjugates
Source: Int J Mol Sci. 2023 Sep 12;24(18):13971. doi: 10.3390/ijms241813971 (PMC10531372; doi:10.3390/ijms241813971)
Supplement: Supplementary file 1 [file ijms-24-13971-s001.zip › ijms-2595981-supplementary.pdf]

Supplementary Information

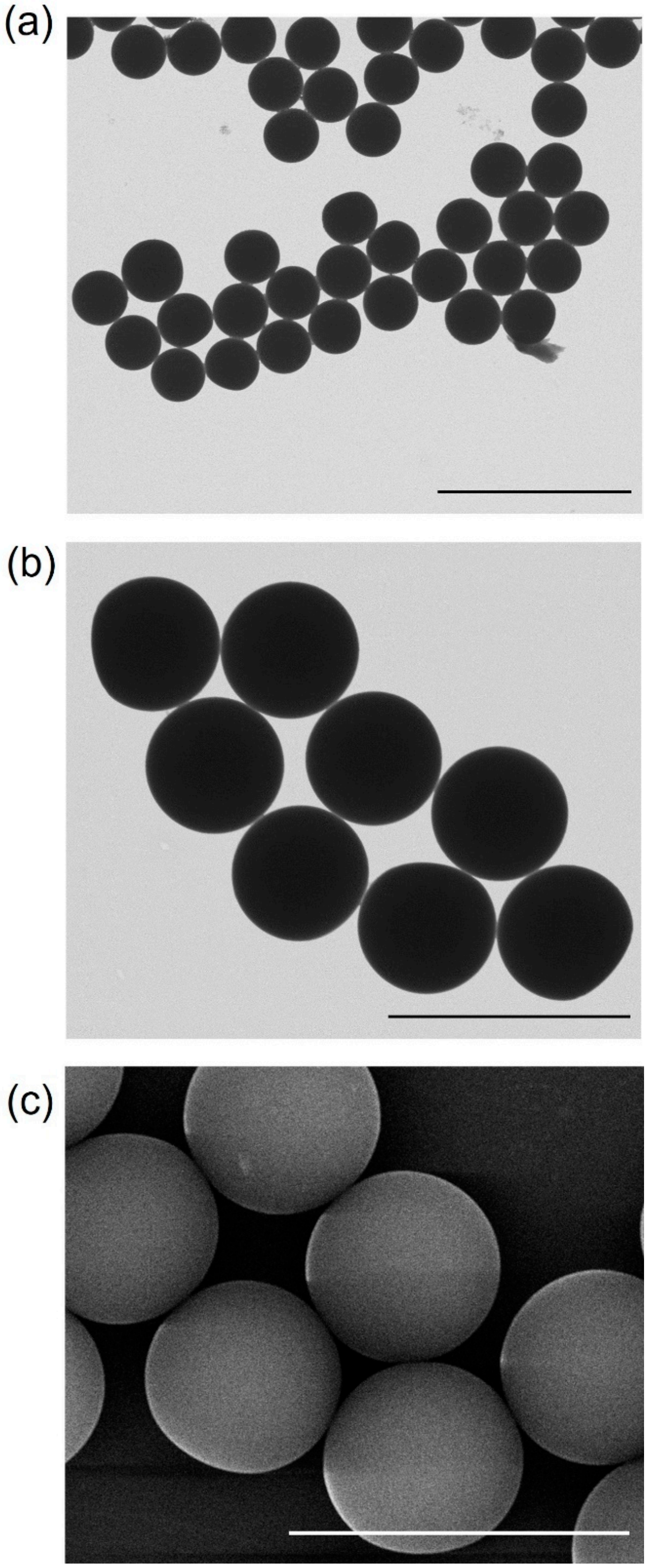

Figure S1. Electron microscopy of anti-Lipid A particles: (a) STEM image at 9,992 x magnification (scale bar: 10 µm), (b) STEM image at 24,938 x magnification (scale bar: 5 µm), and (c) SEM image at 35,000 x magnification (scale bar: 5 µm).

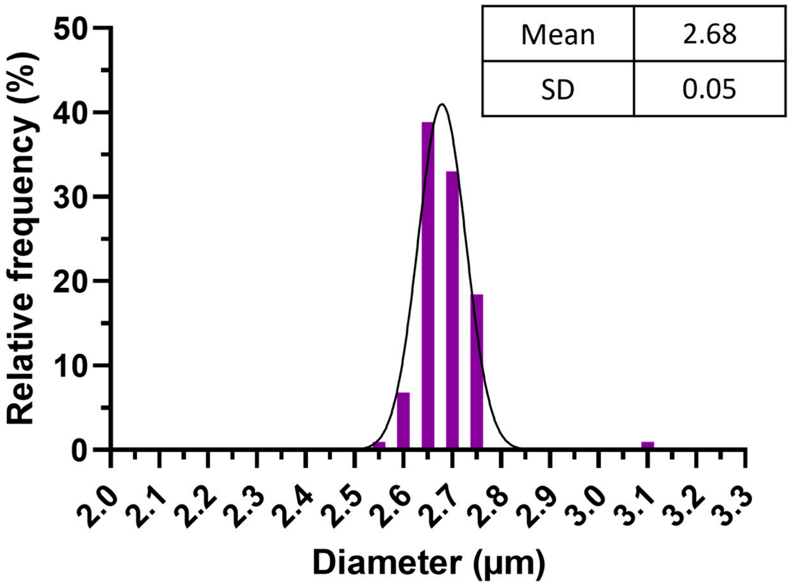

Figure S2. Size distribution of anti-Lipid A particles based on STEM images. More than 100 particles were analyzed using ImageJ.

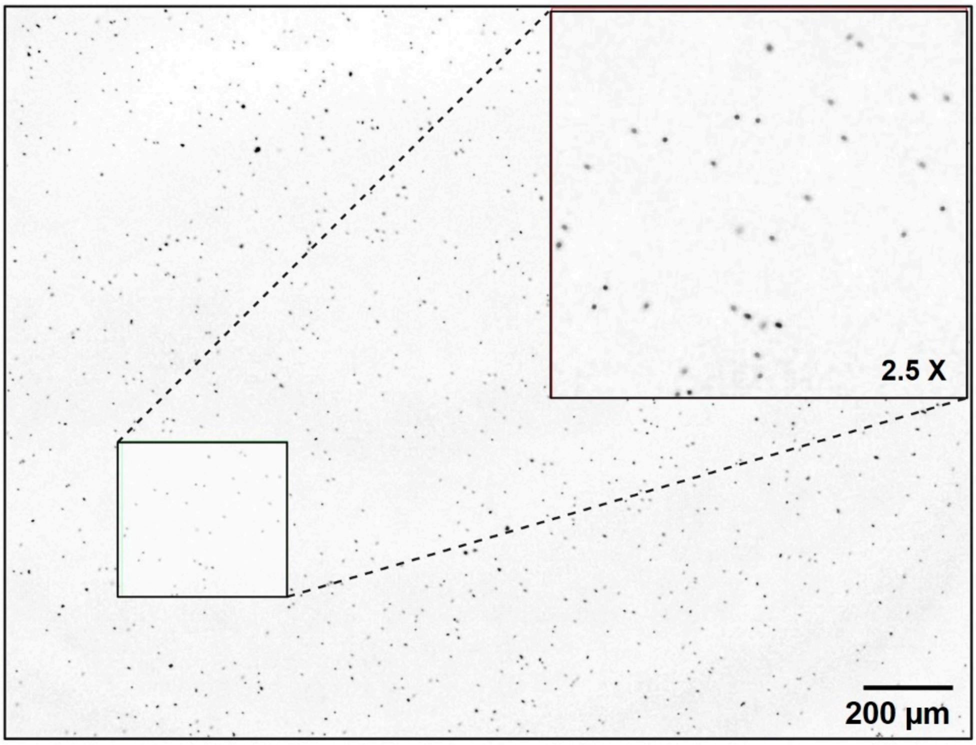

Figure S3. Brightfield image of anti-Lipid A particles, including a zoomed area. Image of anti-Lipid A particles in PBS-0.03% Pluronic F-68, acquired with Countess II Cell Counter from Thermo Fisher Scientific.

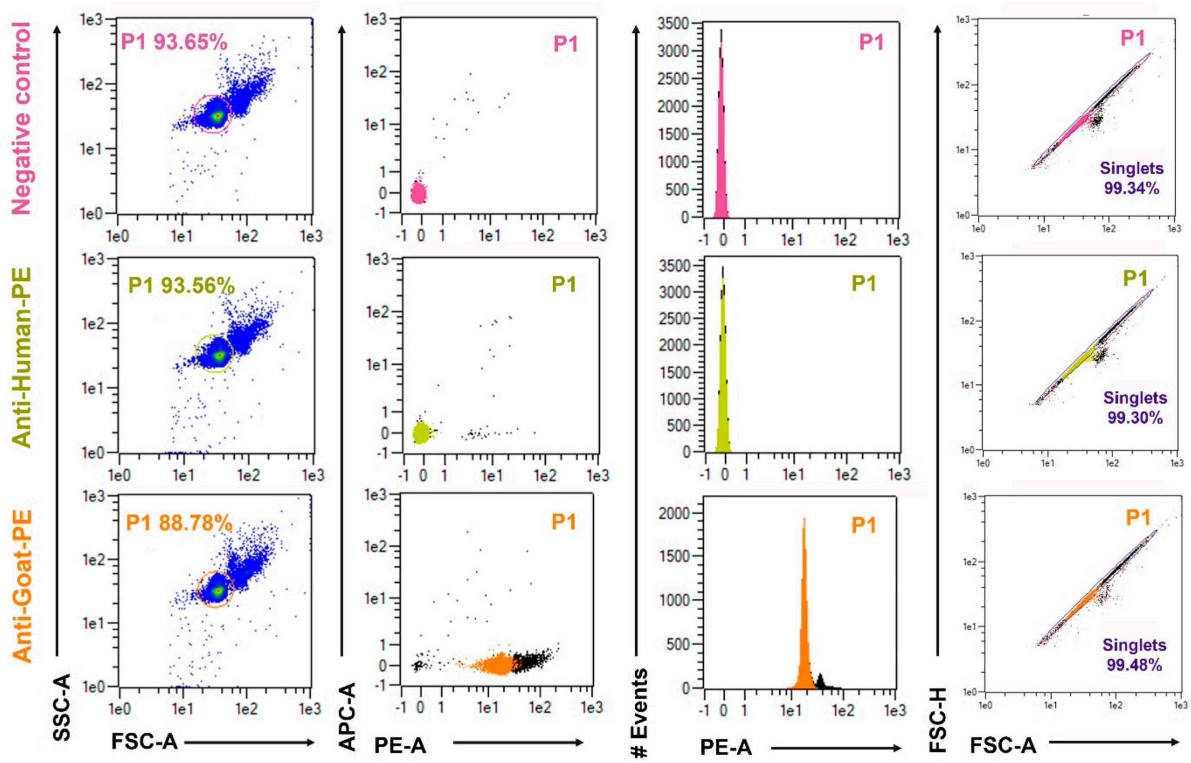

Figure S4. Anti-Lipid A particles analyzed by flow cytometry, using a MACSQuant 10 instrument. P1 represents the main population. The top row corresponds to anti-Lipid A particles without any fluorescent secondary Ab staining. Middle row are anti-Lipid A particles stained with fluorescent anti-Human-PE secondary Ab, which does not recognize the Ab on the particle surface. Bottom row are anti-Lipid A particles stained with fluorescent anti-Goat-PE secondary Ab, which recognizes the Ab on the particles. All three samples show high singlet counts, and only the samples stained with anti-Goat-PE show a fluorescent signal in the B2/PE channel. This indicates a specific staining confirming the presence of anti-Lipid A Ab on the surface of the particles.

(a)

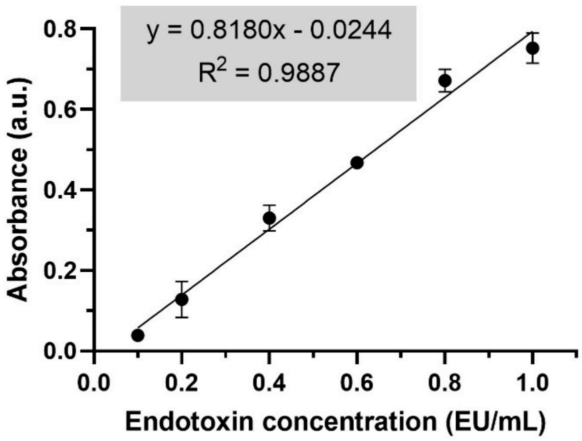

(b)

Sample	Absorbance	Concentration (EU/mL)	Dilution	Concentration corrected (EU/mL)	% Recovery	% Removal
Step 4 saturation curve	0.0245	< 0.1	1	< 0.1	0.0010	99.9990
Step 5 saturation curve	0.0845	0.1329	1	0.1329	0.0013	99.9987

Figure S5. LAL assay. (a) Representative LAL calibration curve used for the experiments presented in this study. Calibration curves were performed for each experiment. (b) Representative data obtained in the LAL test. All results were adjusted by the corresponding dilution factor. The recovery and removal percentages are calculated as shown in Equation S1 and Equation S2.

Equation S1

$$\% \text{ Recovered (LAL)} = \frac{\text{Endotoxin concentration (EU/mL) } \mathbf{SN}}{\text{Initial endotoxin concentration (EU/mL)}} \text{ x } 100$$

Where the Initial endotoxin concentration is 10,000; 1,000 or 100 (EU/mL) depending on the sample.

Equation S2 – General equation for percentage removal (for LAL, NTA and HEK Blue)

$$\% \text{ Removed} = 100 - \% \text{ Recovered}$$

Equation S3

$$\% \text{ Recovered (NTA)} = \frac{\text{\# Particles/frame in } \mathbf{SN}}{\text{\# Particle / frame LPS } \mathbf{Reference}} \text{ x } 100$$

Equation S4

$$\% \text{ Recovered (HEK Blue)} = \frac{\text{Endotoxin (EU/mL) in } \mathbf{SN}}{\text{Endotoxin (EU/mL) in } \mathbf{Sample\ Control}} \text{ x } 100$$

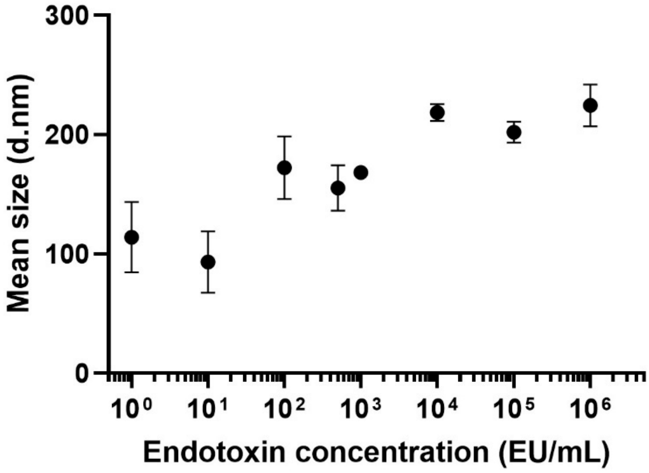

Figure S6. Mean size of supramolecular structures of LPS determined by NTA. LPS from *E. coli* O111:B4 diluted in water. A sample of 1 EU/mL already presents supramolecular structures in NTA.

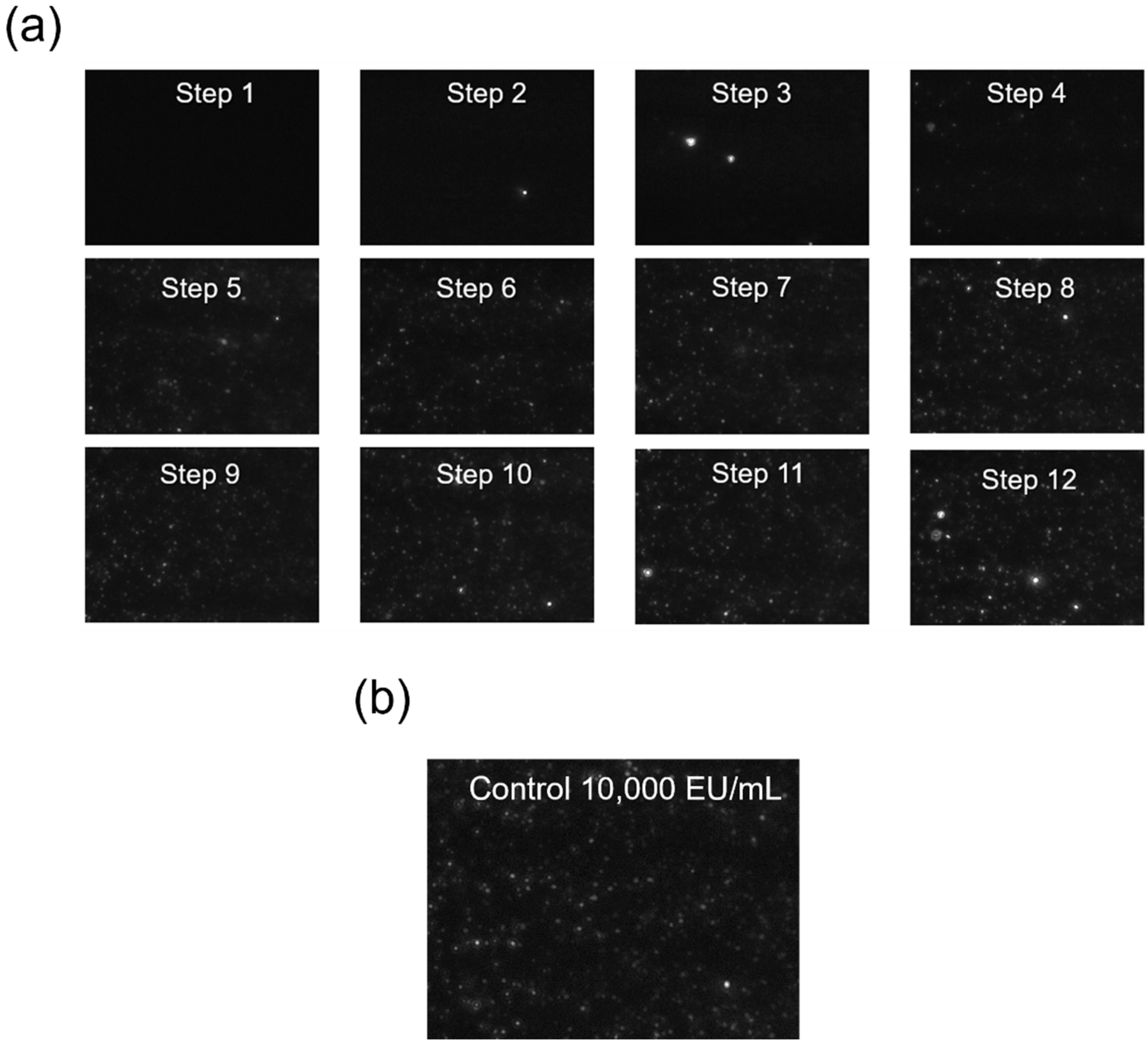

Figure S7. Representative frames from movies generated by NTA showing the increasing number of particles (a) after each removal step of 10,000 EU in the saturation curve with anti-Lipid A particles and (b) the control with 10,000 EU/mL.

From NanoSight report

Results

Stats: Merged Data

Mean:	166.0 nm
Mode:	133.6 nm
SD:	50.0 nm
D10:	117.0 nm
D50:	152.9 nm
D90:	226.8 nm

Stats: Mean +/- Standard Error

Mean:	166.2 +/- 1.9 nm
Mode:	133.4 +/- 1.8 nm
SD:	50.0 +/- 1.5 nm
D10:	117.1 +/- 0.4 nm
D50:	153.1 +/- 1.1 nm
D90:	224.4 +/- 6.0 nm
Concentration:	6.31e+009 +/- 3.00e+008 particles/ml
	320.1 +/- 15.2 particles/frame
	297.8 +/- 9.7 centres/frame

Replicate #	Sample	# Particles/frame	% Recovered	% Removed
1	Step 1	No supramolecular structure found	0	100
	Step 2	1	0.3	99.7
	Step 3	5.7	1.8	98.2
	Step 4	47.8	14.9	85.1
	Step 5	201.6	63.0	37.0
	Step 6	263.6	82.3	17.7
	Step 7	225.7	70.5	29.5
	Step 8	275.7	86.1	13.9
	Step 9	266.8	83.3	16.7
	Step 10	268	83.7	16.3
	Step 11	303.4	94.8	5.2
	Step 12	317.7	99.3	0.7
	LPS 10,000 EU/mL Reference	320.1		

Figure S8. Exemplary NTA report with the average number of particles/frame, which was compared to the control to calculate the LPS removal efficiency (%), for calculations check Equations S2 and S3. Three independent replicates were acquired for all points and were used for the construction of the saturation curve.

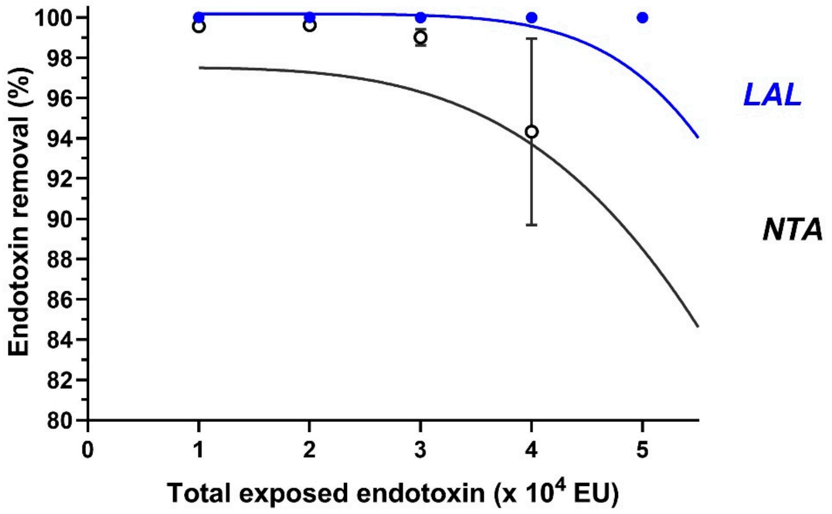

Figure S9. Zoomed area of the saturation curve from Figure 2b showing the first points obtained after exposing 5 mg anti-Lipid A particles to repeated cycles of 10,000 EU of LPS from *E. coli* O111:B4 in water. The removal performance was higher than 98% for the 3 first cycles measured by NTA and 5 cycles measured by LAL.
